# A case report of endometrial adenocarcinoma with leptomeningeal metastases

**DOI:** 10.1002/ccr3.4791

**Published:** 2021-09-21

**Authors:** Danial Fazilat‐Panah, Mansoureh Dehghani, Nahid Ahmadi, Masoumeh Karimi, Sakineh Soleimani Varaki, Ali Emadi Torghabeh, Hamide Mahmoudi, Zahra Keshtpour Amlashi, Mohammadreza Saghafi, Maedeh Alsadat Fatemi, Amir Shokri Bousjin

**Affiliations:** ^1^ Cancer Research Center Babol University of Medical Sciences Babol Iran; ^2^ Cancer Research Center Mashhad University of Medical Sciences Mashhad Iran; ^3^ Department of Radiotherapy and Oncology Faculty of Medicine Mashhad University of Medical Sciences Mashhad Iran; ^4^ Hamadan University of Medical Sciences Hamadan Iran; ^5^ Qazvin University of Medical Sciences Qazvin Iran; ^6^ Cancer Research Center Qazvin University of Medical Sciences Qazvin Iran

**Keywords:** endometrial adenocarcinoma, leptomeningeal metastases, solid tumors

## Abstract

In patients with a history of solid tumors, any new onset of neurological symptoms should be assessed for central nervous system involvement even in rare cases such as gynecological malignancies that nervous system involvement is a rare event.

## Graphical abstract

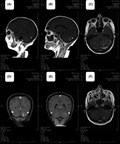

## 1 INTRODUCTION

Genitourinary cancers rarely cause leptomeningeal metastases (LM), and it is even less frequent in endometrial cancers. Any new onset of neurological symptoms should alert the physician to evaluate the patient for central nervous system involvement. Here, we report LM in a patient with metastatic endometrial adenocarcinoma who presented with focal neurological symptoms.

The two inner meninges, the arachnoid and the pia mater, between which circulates the cerebrospinal fluid, are called leptomeninges that can be involved by tumoral cells in patients suffering from malignancies. This condition that has a very poor prognosis and a devastating outcome is named as the leptomeningeal metastases (LM) which is generally a rare condition.^1^, ^2^ The incidence of LM in cancer patients varies with the type of primary cancer as well as the stage of the disease, but it is often estimated to range between 3% and 5%.^3^ Leptomeningeal metastasis resulting from solid tumors most commonly arise from breast, lung cancers, and melanoma.^4^, ^5^ Genitourinary cancers (including ovarian, prostate, bladder, kidney, and cervical cancers), however, rarely cause LM, with only case reports being published. It is anticipated that LM may become more common as patients with cancer in general and GU cancers in particular survive longer with more effective treatments.^6^ The incidence of brain metastases among patients with endometrial carcinoma is 0.6%.^7^ Unfortunately, patients suffering from LM have a poor prognosis leading to a median survival of few weeks to months even after several lines of salvage treatments.^8^ Here, we present a patient with metastatic endometrial carcinoma and focal neurologic symptoms due to LM.

## 2 CASE

**FIGURE 1:**
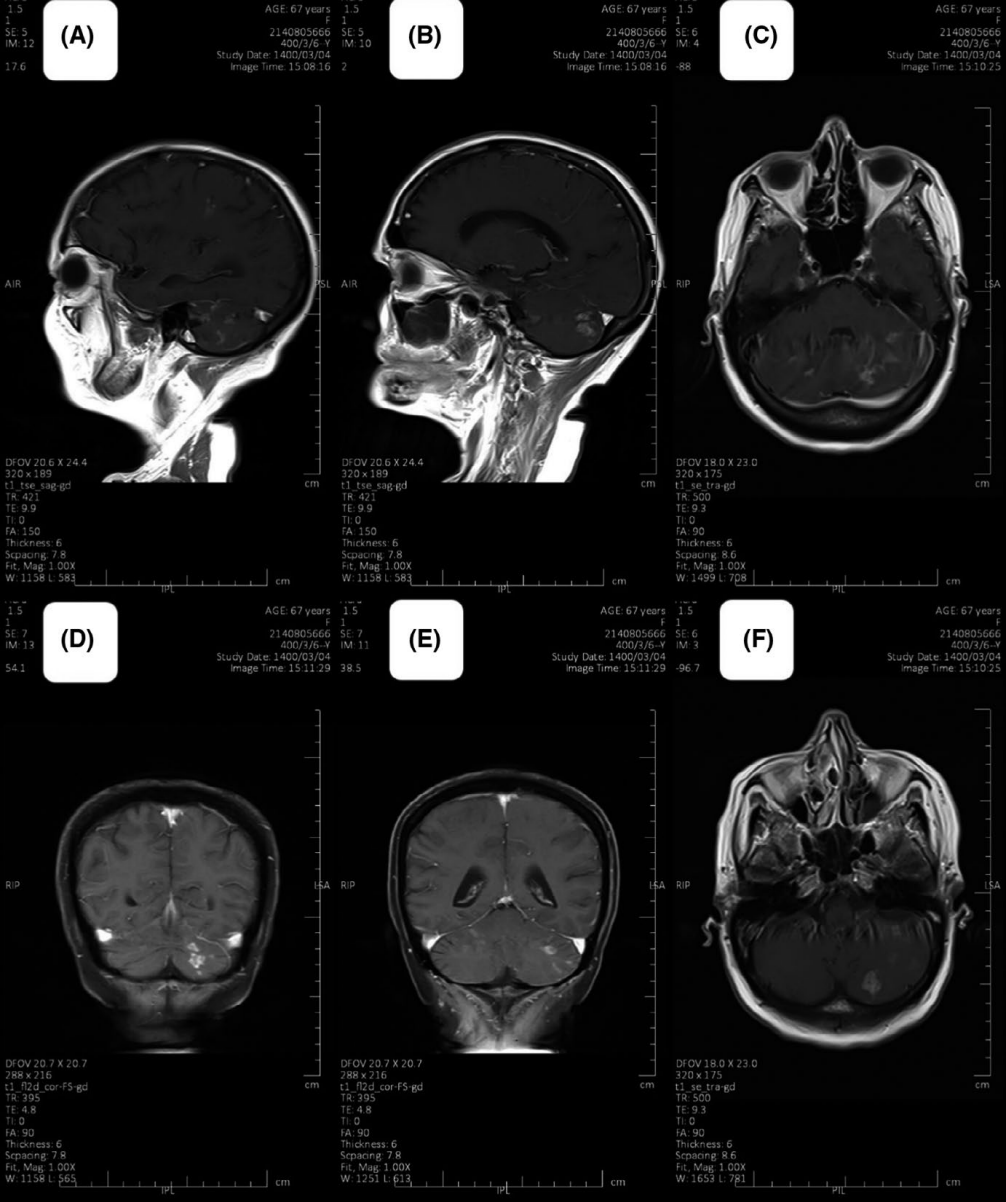
Brain MRI obtained in sagittal plane (A and B), axial plane (C and F), and coronal plane (D and E). On post‐contrast MR images, there are multiple highly enhancive foci at the cortical surface of both cerebellar hemispheres and posterior aspects of both parietal and occipital lobes, which all are suggestive of leptomeningeal metastases

A 67‐year‐old woman with a history of endometrial carcinoma was admitted to the oncology department due to focal neurological symptoms. The only comorbidity other than uterine cancer was controlled hypertension. In the patient's history, pathologic assessment following total abdominal hysterectomy and bilateral salpingo‐oophorectomy demonstrated a high‐grade uterus endometrial adenocarcinoma, which was located at the fundus with deep myometrial invasion (>50%) without involvement of the uterine serosa, the lower segment, and the cervix. Lymphovascular invasion was present, although no lymph node was assessed. With a FIGO stage IB tumor, a multidisciplinary team (MTD) advised a postoperative adjuvant chemoradiotherapy and subsequent chemotherapy due to the primary surgery being non‐oncologic. The patient was treated with the prophylactic whole‐pelvic radiotherapy (45 Gy in 25 fractions) with concomitant weekly carboplatin (area under the curve (AUC) = 2) and paclitaxel (50 mg/m^2^). After that, adjuvant chemotherapy was prescribed using docetaxel (100 mg/m^2^) plus carboplatin (AUC: 7) every 3 weeks for four cycles. After 3 years of follow‐up which all assessments were normal, the patient underwent a spiral chest computed tomography scan (CT) because of progressive shortness of breath and persistent coughs, which revealed extensive pulmonary metastases. A whole‐body CT scan demonstrated no other distant metastasis or local recurrence. A rechallenge by docetaxel plus carboplatin was initiated resulting in complete radiological response and disappearance of the patient's symptoms after six courses of chemotherapy. After 1 year of follow‐up, the patient suffered from dyspnea again, and the CT scan showed a recurrence of pulmonary metastases. At this stage, six courses of paclitaxel (175 mg/m^2^) and carboplatin (AUC: 7) every 3 weeks resulting in complete radiological response once more. After 6 months, the patient was admitted to our department due to focal neurological deficits including myoclonus and paresthesia of the right upper limb. A brain magnetic resonance imaging (MRI) with and without gadolinium revealed multiple highly enhancive foci at the cortical surface of both cerebellar hemispheres and posterior aspects of both parietal and occipital lobes on post‐contrast MRI images, there are which all are suggestive for leptomeningeal metastases (Figure 1). The patient refused to undergo lumbar puncture (LP) for cerebrospinal fluid (CSF) examination.

Whole‐brain radiotherapy (WBRT) to 30 Gy in 10 fractions was delivered to the all parts of brain parenchyma and surrounding meningeal spaces and meninges; subsequently, liposomal doxorubicin 40 mg/m^2^ was prescribed every 4 weeks. With a follow‐up of 6 months, the neurologic symptoms of the patient were improved significantly. Moreover, the patient was disease‐free based on a contrast‐enhanced whole‐body CT scan and brain MRI in the sixth month.

## 3 DISCUSSION

The diagnosis of LM is rare, and it is even less frequent in genitourinary tract cancers. Most cases of LM in gynecologic cancers have been described in ovarian cancer, with only a few reports associated with cervical or uterine cancer. In the future, LM may be increasingly diagnosed. One reason is the enhanced ascertainment of the condition due to improvements in diagnostic techniques. Additionally, due to the continued improvements in the understanding of the biology and treatment of these cancers and the associated longer survivals, the incidence of these rare metastatic conditions may arise.^6^ Leptomeningeal metastases are usually a late manifestation of systemic disease and most often occur in patients after extensive therapy with surgery, radiation, and chemotherapy.^9^ The most common presenting symptoms of LM are findings related to increased intracranial pressure, such as headache, nausea, vomiting, and gait disturbances. Nuchal rigidity is present in only 15% of cases. Classic cerebral signs such as hemiparesis and aphasia are uncommon. However, because any part of the CNS may be involved in LM, patients may present with a variety of symptoms.^10^ New onset of neurological symptoms should alert the physician to evaluate the patient for any CNS involvement.^11^ This explains our patient's rare manifestations with focal neurological deficits including myoclonus and paresthesia of the right upper limb. It highlights the key point that high‐clinical suspicion is the most important step to achieve an early diagnosis.

Tumor metastasis to the meninges typically occurs by one of the four mechanisms: meningeal seeding from preexisting hemispheric CNS metastases, direct extension from subdural or epidural tumor, direct extension from sights outside but adjacent to the CNS, and hematogenous spread.^12^

The definitive diagnosis of LM is only done by documenting the presence of malignant cells in the CSF, but in one‐third of the patients, the CSF cytology is not diagnostic, and repeated CSF cytological examination would be necessary.^12^, ^13^ Diagnosis via a cerebral MRI has been reported but only MRI with Gd contrast can reveal the pathology, while cerebral CT or MRI without contrast are negative most of the time.^14^

Treatment of LM is palliative and the aim is to prevent progression or development of new neurological deficits and improve quality of life. Typical treatment applied by clinicians for LM includes external beam radiation and systemic and intra‐CSF chemotherapy.^15^, ^16^, ^17^ The present case was treated by WBRT followed by systemic chemotherapy and is still asymptomatic and without disease progression in short‐term follow‐up. However, there is a definite need for longer follow‐up and more evaluation, especially analyzing case series as the prevalence increases, to establish a standard treatment or approach for this rare condition.

Considering new evolution in the radiotherapy techniques enabling radiation oncologist to delineate to the tumor and spare the organ at risks more accurately leading to the better tumor control by higher radiotherapy doses and lower dose to the normal tissues, using these techniques in the patients with LM without involvement of bran parenchyma might help to improve the results.^18^, ^19^ Besides, there are promising new data on the underlying molecular mechanisms of different cancers that may improve the outcome of patients with LM through introducing new drugs and treatment modalities.^20^, ^21^, ^22^, ^23^, ^24^

## 4 CONCLUSION

Leptomeningeal metastases is an extremely rare manifestation in endometrial cancers. Early diagnosis may be achieved by high‐clinical suspicion and special consideration of any newly onset neurological symptoms.

## CONFLICTS OF INTEREST

None declared.

## AUTHOR CONTRIBUTIONS

All authors contributed equally.

## ETHICAL APPROVAL

The study was approved by Babol University of medical Sciences. The study conforms to recognize the standards of Declaration of Helsinki.

## CONSENT

An informed written consent form was obtained from patient. He agreed that his information can be published anonymous.

## Data Availability

The data sets used and analyzed during the current study are available from the corresponding authors per request.
